# Librarian: An Open-Access Web Application for High-Resolution Mass Spectral Library Assembly

**DOI:** 10.3390/metabo16060433

**Published:** 2026-06-22

**Authors:** Jacob Ahlberg Weidenfors, Bénilde Bonnefille, Stefano Papazian

**Affiliations:** 1 Unit of Integrative Metabolomics, Institute of Environmental Medicine, Karolinska Institutet, 17177 Stockholm, Sweden; jacob.ahlberg.weidenfors@ki.se; 2 Science for Life Laboratory, Department of Environmental Science, Stockholm University, 11418 Stockholm, Sweden; benilde.bonnefille@aces.su.se; 3 National Facility for Exposomics, Science for Life Laboratory, Stockholm University, 17165 Solna, Sweden

**Keywords:** open-source, spectral database, mass spectrometry, HRMS, metabolomics, exposomics, MassBank, MS-DIAL, NTA, FAIR

## Abstract

**Background**: Confident chemical annotation in nontarget small-molecule mass spectrometry critically depends on the availability of high-quality tandem mass spectral (MS^2^) reference libraries. While community efforts have driven significant expansion of open-access repositories, technical challenges in assembling standardized, metadata-rich records continue to limit broader participation, underscoring the need for improved computational tools to assist contributors. **Methods**: To promote the creation and sharing of standardized reference MS^2^ spectral records, we have developed Librarian, a free, open-access web application designed for rapid and scalable assembly of high-resolution MS^2^ libraries. Librarian integrates automated retrieval and harmonization of chemical identifiers and metadata from PubChem, compound mixture design for high-resolution mass spectrometry (HRMS) acquisition, and assembly of curated MS^2^ spectra into repository-ready records compatible with public spectral databases. **Results**: Through a simple in-browser interface, Librarian offers a flexible end-to-end workflow compatible with popular open-source pre-processing tools to lower technical barriers and facilitate broader community participation in library development. As a demonstration, we used Librarian to create and deposit a spectral library comprising over 1500 new MS^2^ records into MassBank, which was further applied in retrospective analysis of environmental datasets. **Conclusions**: Librarian streamlines the creation of standardized, metadata-rich and repository-ready MS^2^ reference records. Addressing a key bottleneck in community spectral library development and sharing, Librarian supports the continued growth of open-access resources for metabolomics, exposomics, and environmental mass spectrometry. The Librarian web application is publicly accessible via the SciLifeLab Serve platform.

## 1. Introduction

Nontarget analysis (NTA) of small molecules using high-resolution mass spectrometry (HRMS) commonly results in the detection of thousands of molecular features, posing substantial challenges for their annotation and identification. In metabolomics, exposomics and environmental research, the acquisition and comparison of tandem mass spectra (MS^2^) with reference library records have become the primary means of achieving confident feature annotation in NTA [1,2,3], driving rapid expansion of public spectral resources in recent years [4,5,6,7]. The growth of public repositories has been intimately linked with notions of open science as formulated in the FAIR principles [8] and has seen the parallel development and adoption of standardized record formats to provide a framework for open and reliable data sharing.

Despite the rapid growth of public spectral repositories, demand for reference spectra remains high to further improve annotation rates in NTA. Besides the need to expand database coverage to novel compounds, repositories and users recognize the value of accessing multiple reference spectra per compound recorded under different conditions, e.g., instrumentation, resolution, ionization mode and collision energies, to match a given experimental spectrum. As a result of considerable community efforts in open spectral sharing, public repositories such as MassBank Europe [5] (MassBankEU, henceforth simply referred to as MassBank [9]), and Global Natural Products Social Molecular Networking (GNPS [6,10]) currently host MS^2^ records for several hundred thousand compounds, acquired across diverse instruments and experimental conditions. The major public repositories notably differ in their approach to sourcing, curation, and hosting of reference spectra. Of the two most prominent platforms, GNPS emphasizes aggregation of reference data from multiple sources with relatively limited curation and formatting constraints to maximize library size, whereas MassBank exclusively hosts records directly submitted by users in a standardized format.

The MassBank database is cross-integrated with GNPS and other major public repositories such as MassBank North America [11], the US EPA CompTox Dashboard [12,13], NORMAN Database System [14,15], RforMassSpectrometry [16], and PubChem. As of release 1 May 2025, MassBank consists of 119,845 unique spectra representing 18,529 unique compounds [5]. Emphasizing data quality and FAIR principles, MassBank requires contributors to provide extensive chemical and acquisition-related metadata alongside the spectral data, which are integrated and stored as a structured, human- and machine-readable text file with standardized field labels. An effective gold standard for open-access spectral records, the requirements of the MassBank format do however impose non-trivial demands on contributors during the process of record assembly. Whereas MS^2^ data acquisition and analysis are routine activities for analytical laboratories, converting spectral data into repository-ready records can be challenging for researchers with limited informatics expertise and may restrict community participation in public repository projects. Consequently, there is a need for accessible tools that simplify the recording, assembly, and sharing of spectral reference data to accelerate public repository growth.

Alongside their core functionality, popular open-source pre-processing software for MS-based metabolomics such as MZmine [17,18,19] and MS-DIAL [20,21] include modules for exporting MS^2^ spectral data in common formats, but support for end-to-end spectral library creation, assembly, and deposition is currently limited in flexibility and scope (Table 1). Recent MZmine releases introduced metadata retrieval and library generation workflows developed within the scope of the MSnLib project [19], which however rely on adjunctive Python scripts and do not currently support exporting of MassBank-format records. Although the MassBank consortium provides the R-based package RMassBank with functionality for MS^2^ extraction, fragment annotation, metadata retrieval, and assembly of MassBank-format records [22,23], its workflow is restricted to the R programming environment and lacks direct export support for other spectral formats widely used in third-party software and computational workflows, such as .msp (e.g., NIST, MS-DIAL) and .mgf (e.g., GNPS, SIRIUS). To our knowledge, the only other existing web-based tool specifically designed for spectral library creation is Curatr (EMBL) [24], but it is currently unavailable pending service migration (personal communication).

To broaden community participation, there is a clear need for platform-agnostic tools that lower technical barriers while enabling flexible and interoperable workflows. To address these limitations, we developed *Librarian*, a web application that supports large-scale data acquisition, metadata retrieval, and record assembly compliant with the metadata and formatting requirements of MassBank. Librarian is designed to be simple and user-friendly, providing a collection of modular resources for a complete MS^2^ library creation workflow. Librarian automates retrieval and harmonization of chemical identifiers and metadata via PubChem, generates MS^1^ accurate masses for targeted acquisition, supports design of compound mixtures, and flexibly assembles MS^2^ spectra and metadata into standardized reference library formats (.txt and .msp) ready for deposition in public repositories. To promote accessibility and interoperability, Librarian is compatible with spectral data exported from widely used open-source pre-processing software and allows extensive user control over the final library assembly steps, including options for adding additional compound metadata information such as retention time indexing (RTI) [25] and chemical class ontologies (ClassyFire) [26]. In combination, the Librarian modules and utilities provide a complete workflow for assembling spectral libraries for deposition and reuse via public repositories and in retrospective NTA applications. Finally, we demonstrate our use of Librarian to create and deposit a 1200-compound pharmaceutical spectral library in MassBank and subsequently apply it in retrospective NTA of publicly available environmental datasets. The Librarian web application is publicly accessible via the SciLifeLab Serve platform (https://librarian.serve.scilifelab.se).

## 2. Materials and Methods

### 2.1. Implementation

**Figure 1:**
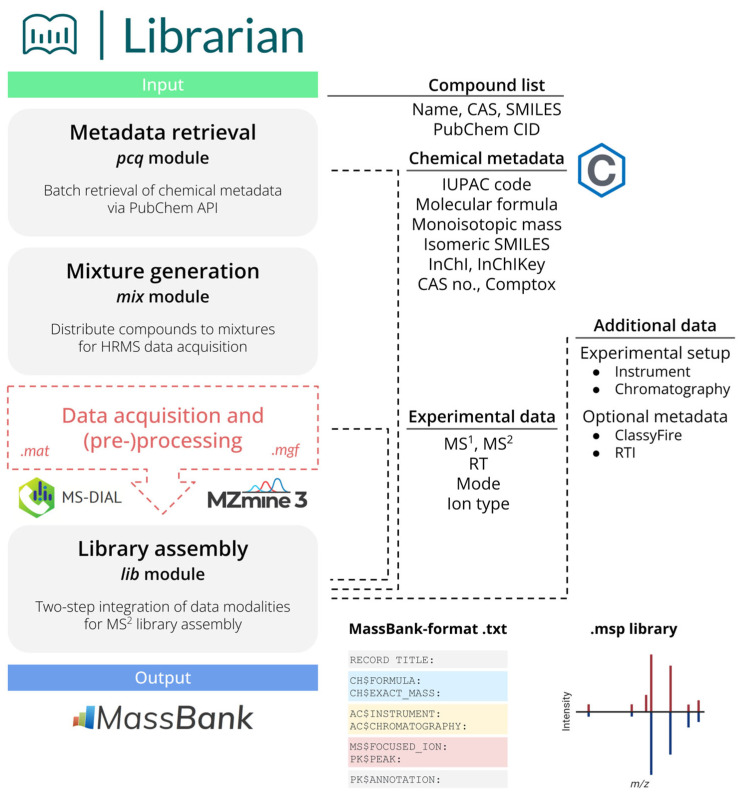
Overview of the Librarian workflow. Librarian combines functionality from three core modules for rapid, scalable, and flexible assembly of spectral libraries. Starting from an input list of compounds, the workflow retrieves chemical metadata from PubChem (pcq module), designs mixtures for data acquisition (mix module) and integrates externally processed spectral (MS^2^) data with metadata into a complete MassBank-formatted spectral library (lib module). Librarian is publicly accessible via the SciLifeLab Serve platform (https://librarian.serve.scilifelab.se).

Librarian is implemented as a modular web application for streamlined, scalable, and automated execution of tasks covering a complete MS^2^ library assembly workflow (Figure 1). As a starting point, the user only requires minimal information of the chemical compounds to be included in the library, which can be described using common names, CAS registry numbers, SMILES strings, PubChem CIDs, or combinations thereof. In each module, user inputs (e.g., in-browser data entry, or spreadsheet uploads) are then processed to generate defined outputs (e.g., annotated spreadsheets and visual summaries), allowing stepwise progression which easily guides the user throughout the workflow.

First, the PubChem query (“pcq”) module interfaces with PubChem servers to retrieve comprehensive chemical metadata (e.g., chemical formula, monoisotopic mass, isomeric SMILES) for each compound entry and returns results in spreadsheet format. Optionally, the pcq module output can be passed to the Mixture design (“mix”) module, which distributes compounds into mixtures based on their physicochemical properties to assist in experimental design for HRMS MS^2^ data acquisition. For this purpose, the mix module also calculates expected accurate masses, ion species, and charged adducts for each ionization mode. Following data acquisition and pre-processing using any Librarian-compatible software (e.g., MS-DIAL, MZmine, OpenMS), the Library assembly (“lib”) module integrates processed spectral data with chemical and instrumental metadata (e.g., instrument type, acquisition parameters) to generate a spectral library conforming to MassBank formatting standards. Record assembly via the lib module is extensively customizable by the user. A detailed description of the Librarian workflow modules is given in the following sections.

### 2.2. Software Framework

Source code for Librarian is written in Python (version 3.12.3). The Librarian web application is built using the open-source Python framework Streamlit (version 1.45.1 [27]), adapting a command line (CLI)-based workflow to web-based interaction. Besides standard packages, functions from several chemistry- and MS-oriented packages are incorporated into the workflow, notably RDKit, PubChemPy, pysplash and IsoSpecPy. While Librarian module functions can also be accessed via CLI, the web application of Librarian is recommended for ease of use. All source code is freely accessible via the Librarian GitHub repository (https://github.com/jahlwe/streamlit-librarian).

### 2.3. Input Requirements

Librarian is designed to require minimal starting input from users. To initialize the workflow, users provide a list of compounds intended for MS^2^ acquisition to the PubChem query (pcq) module. In the web application, compound lists may be entered directly in-browser or uploaded (.csv or .xlsx). Each compound must be described by at least one of either common name, CAS registry number, SMILES string, and/or PubChem CID.

### 2.4. Metadata Retrieval via PubChem Query (pcq Module)

**Figure 2:**
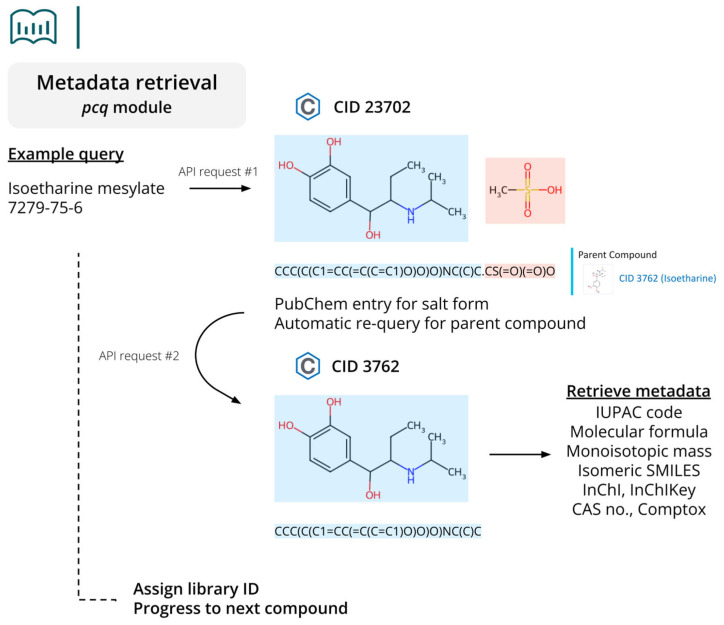
Metadata retrieval via PubChem query (pcq module). Users initialize the workflow by submitting compound lists to the PubChem query (pcq) module via browser entry or .csv/.xlsx upload using chemical names, CAS numbers, SMILES strings, or PubChem CIDs, although unambiguous identifiers (CID, CAS, or SMILES) are preferred to minimize incorrect matches. Retrieved records are parsed to extract MassBank-relevant metadata and additional identifiers/cross-references. For salt forms (e.g., isoetharine mesylate), Librarian automatically detects and resolves parent compounds from SMILES patterns and PubChem annotations before assigning a library identifier for downstream workflow integration.

The PubChem query (pcq) module retrieves chemical metadata using the Python package PubChemPy (Figure 2). Queries are executed via programmatic requests to the PubChem REST API, employing both PubChemPy-native functionality and custom implementations for retrieving additional identifiers. From returned data, metadata required by the MassBank format are extracted, including molecular formula, monoisotopic mass, and isomeric SMILES. Additional identifiers not explicitly required by MassBank specifications (e.g., InChIKey) and cross-references to external databases (e.g., CAS registry numbers, CompTox Dashboard [12,13]) are also extracted and stored, when available. For compounds queried as salt forms, which typically do not resolve the corresponding parent compound, Librarian interrogates returned records for (1) the presence of a dot (“.”) in the isomeric SMILES indicative of salts or mixtures and (2) the presence of explicit “Parent Compound” annotations within the PubChem data structure. Detection of a parent compound triggers automatic re-query for the corresponding neutral species, eliminating the need for manual curation or prior removal of salts from the input compound list. When chemical names (e.g., IUPAC) used for metadata queries return multiple matching PubChem entries, metadata is extracted from the first (best-match) record returned by PubChem.

It should be noted that the pcq module is inherently dependent on the PubChem API, and its output is therefore contingent to the information retrieved from PubChem. As a benchmarking exercise, we manually collected and compared the correct reference PubChem CIDs for 1200 FDA-approved pharmaceuticals (see later Section 3.1 and Supplementary Information S1–S4 for details) versus the CIDs automatically retrieved by Librarian using names, CAS registry numbers, and SMILES obtained from our chemical supplier. We found reporting CID error rates to be low for queries based on either CAS (0.8%) or compound name (1.3%); a larger error rate (8.2%) was observed for queries based on SMILES, mainly caused by improper SMILES notations from the chemical supplier (e.g., misplaced double bonds, incorrect cations, etc.). To minimize incorrect matches on PubChem, we recommend that users rely on unambiguous identifiers whenever possible, such as CID, CAS registry numbers, or accurate SMILES strings. However, when using SMILES, users should be aware that poorly curated queries may still result in successful but erroneous metadata retrieval.

Thanks to the high capacity of PubChem servers, queries performed via Librarian are robust, assuming a stable internet connection. A representative query of 1000 compounds using the Librarian web application was completed in approximately 20 min (internet speed at 50 Mbps). To handle failed queries efficiently, compounds are flagged to ensure that only unresolved entries are re-queried following corrections to either in-browser entries or the uploaded spreadsheet. In the latter case, the output sheet with partially complete data should be downloaded, failed query inputs corrected, and this sheet re-uploaded. As an additional feature, along with physicochemical metadata from PubChem, toxicological metadata can be retrieved from the EPA CompTox Dashboard servers by selecting variables of interest in the Parameters menu. Although primarily intended for use within the Librarian workflow, browser-based batch retrieval of chemical metadata provides a standalone utility for large-scale applications.

### 2.5. Mixture Design (Mix Module)

**Figure 3:**
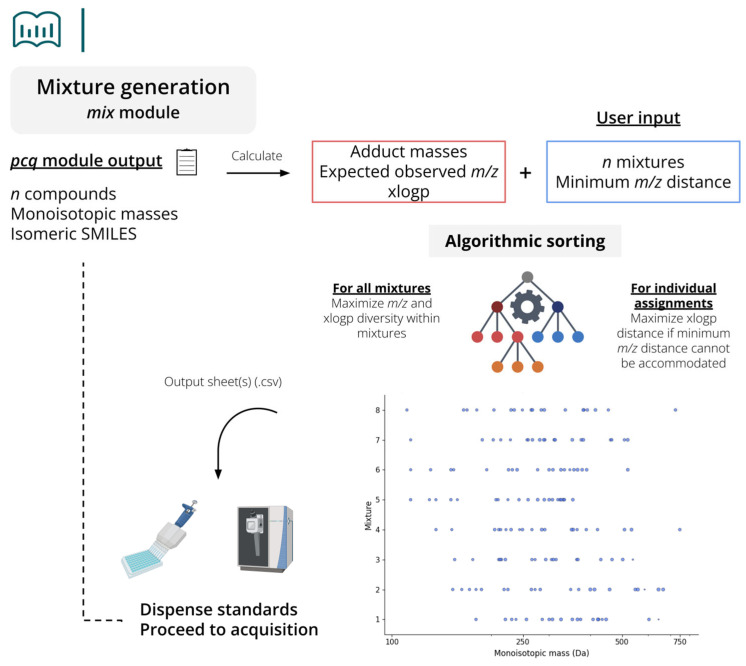
Compound distribution via Mixture design (mix module). The module algorithm assigns compounds to mixtures using calculated *m*/*z* and xlogP to maximize mass diversity and reduce co-elution and ion competition during RPLC-HRMS MS^2^ acquisition. The sorting algorithm is constrained by a user-defined number of mixtures and minimum *m*/*z* separation tolerance within each mixture. Mixture assignments are returned in a spreadsheet (.csv) format.

Following metadata retrieval, the optional mix module helps to design a mixture distribution scheme for efficient HRMS data acquisition (Figure 3). Using the chemical metadata retrieved via PubChem, the mix module compiles mass-to-charge ratio (*m*/*z*) calculated for several commonly observed adducts in liquid chromatography–electrospray ionization (LC-ESI) and estimates compound polarity (xlogP) through RDKit using the atom-based approach of Crippen [28]. An expected *m*/*z* in positive ESI mode—typically, the calculated [M + H]^+^ ion—is determined for each compound and used to distribute compounds to mixtures, with natively charged species handled as exceptions (e.g., [M]^+^). Users define a desired number of mixtures and a minimum *m*/*z* difference between compounds. A deterministic, greedy algorithm is used to assign compounds according to the user-defined constraints. In brief, compounds are iteratively distributed to mixtures such that the minimum *m*/*z* difference is maintained and each mixture contains an approximately equal number of compounds (±1 compound where division is uneven). Besides *m*/*z*, a maximal diversity of chromatographic behavior in each mixture is sought by sorting compounds by xlogP before starting the assignment process. This strategy aims to reduce the likelihood of isomer and isobar co-elution and competition for MS^2^ acquisition in data-dependent (DDA) workflows consistent with the relationship between xlogP and retention in reversed-phase LC (RPLC) systems [29]. Through the combined attention to both *m*/*z* and (predicted) chromatographic behavior, mixtures are formulated to allow efficient data acquisition.

For a given set of compounds and user-defined constraints, the algorithm may occasionally be unable to assign compounds without violating the chosen minimum *m*/*z* difference. In such cases, remaining compounds are assigned solely based on xlogP, and placed in mixtures that maximize the xlogP distance from compounds already present in the mixtures while keeping the total number of compounds balanced (e.g., for 25 unassigned compounds and 20 mixtures, two compounds are added to five mixtures and one to the remaining 15). The mix module returns a spreadsheet file (.csv) with *m*/*z* values for several ion species (both positive and negative mode), xlogP values, and the mixture assignments. Visual and descriptive summaries of the mixture distribution results can also be downloaded by the user for a quick review of the compound pools under the selected parameters.

### 2.6. Library Assembly (Lib Module)

**Figure 4:**
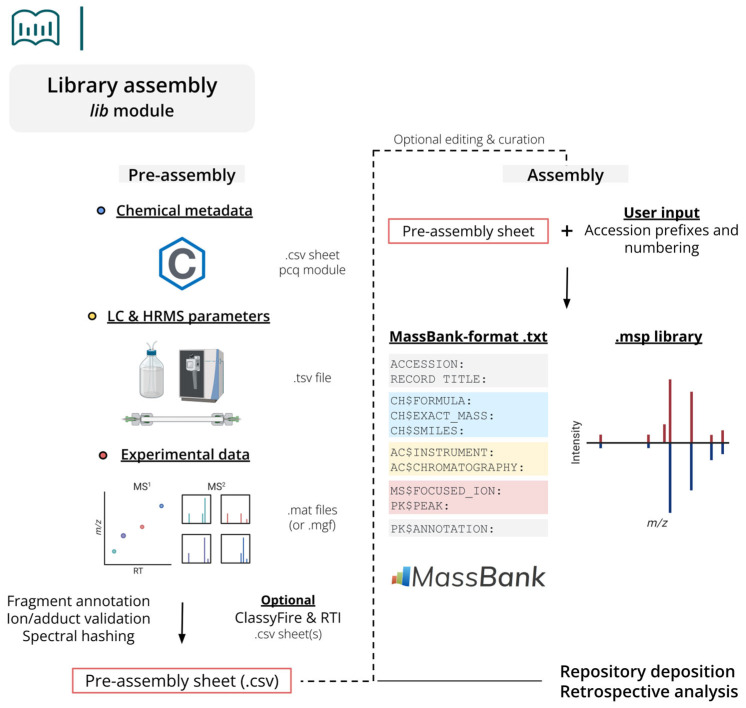
Library assembly in the Librarian workflow (lib module). Library assembly proceeds in two stages. In the pre-assembly submodule, chemical and instrumental metadata, and acquired HRMS data are merged into a “pre-assembly sheet” (.csv). Fragment annotation, ion/adduct validation, spectral hashing is also performed here. In the assembly submodule, the pre-assembly output sheet and a user-defined accession prefix and numbering scheme is processed to create MassBank-formatted (.txt) records and library (.msp) files. Assembly is performed separately per ionization mode.

The lib module integrates data for all compounds and assembles the finalized spectral library. After acquiring and pre-processing data through any of the Librarian-compatible platforms (MZmine, MS-DIAL, OpenMS), the exported spectral data is uploaded together with previous Librarian outputs (Figure 1). The assembly process is performed separately for positive and negative ionization mode data (Figure 4) and consists of two stages: pre-assembly and assembly.

During pre-assembly, all input data is consolidated into a single spreadsheet for use in the subsequent assembly submodule. Minimal inputs for pre-assembly include: (1) a pcq output sheet containing chemical metadata for all compounds; (2) a tab-delimited (.tsv) file specifying LC-HRMS experimental parameters (e.g., instrument, ionization, resolution, collision energy, etc.) and user contributions (authors and affiliations, license and copyright)—a template is available for download via the web application (and is included when downloading the Librarian distribution); (3) spectral data exported from a third-party software (e.g., MS-DIAL, MZmine, OpenMS). If available, retention time indexing (RTI) [24] and chemical ontology data (ClassyFire) [25] can be included by uploading separate .csv files; it should be noted that the latter are automatically provided by MassBank since release 1 May 2025 [5].

To increase flexibility, Librarian provides a utility function to convert the common .mgf format into Librarian-compatible .mat files. Besides the MS^2^ spectrum, information such as precursor *m*/*z* and ion type (see also output from mix module) should be provided in data exported from the chosen pre-processing software. To ensure that data is used correctly, its transfer across utilities and modules (e.g., format conversion, or pre-assembly sheet generation) is customizable via the Parameters interface in the web application. Parameter settings further allow users to include project- or compound-specific data (e.g., collision cross-section values) in the final records.

Beyond data integration, the pre-assembly submodule also optionally performs automated annotation of tentative MS^2^ fragment substructures through a recursive algorithm that generates candidate fragments bounded by the molecular formula, and matches these with experimental fragments at a ppm tolerance chosen by the user (10 ppm by default). To limit spurious or unrealistic formula assignments, the double bond equivalent (DBE) count of each candidate is evaluated, and candidates with DBE < 0 are discarded. The DBE, element ratios (ratios of H, N, O, S and P to C), and ppm deviation are stored for each formula matching a given experimental peak within the user-defined ppm tolerance [30]. Candidate formulas are then ranked using a weighted scoring function that integrates these parameters with isotopic envelope matching, quantified by cosine similarity. Higher scores are assigned to candidate formulas that are non-radical species (inferred from the DBE after accounting for adduct contributions), exhibit elemental ratios within expected bounds [30], and show good agreement between observed and theoretical isotopic envelope, when present. Scores are also weighted inversely to the mass error (ppm), normalized to the user-defined tolerance. Formula assignment is considered unequivocal only when a single candidate formula matches an experimental peak. For peaks with multiple candidate formulas, the highest-scoring candidate is assigned. If present, the molecular ion peak is assigned to its corresponding formula prior to scoring, preventing spurious competition from alternative candidates.

In a final step, the pre-assembly submodule validates each record and alerts users to potential errors in the integrated data. The measured precursor *m*/*z* is first compared against the theoretical *m*/*z* of the assigned ion type (adduct), and alternative ion types are suggested where discrepancies are detected. Additional checks evaluate precursor mass error (ppm) deviation, presence of apparent co-isolated ions in the MS^2^ spectrum, whether the base peak lies near the precursor *m*/*z* (potentially indicating poor fragmentation), presence of the molecular ion peak, and fragment annotation coverage (the latter two only when optional fragment annotation has been performed). The validation results are reported in a dedicated column of the pre-assembly output file for users to review.

Completing pre-assembly returns a .csv sheet with all compiled data, which can be manually reviewed or edited before the final library assembly. Finally, spectral library files are created by uploading the pre-assembly sheet to the assembly submodule, along with a user-defined starting accession number and short and full record prefixes. The output consists of MassBank-formatted (.txt) files for each compound and a consolidated library (.msp) file representing the entire dataset.

### 2.7. Utilities and Customization Options

Besides the core modules, Librarian features a set of utility functions that assist in specific steps of the workflow. Moreover, the functions of both modules and utilities are extensively customizable, allowing users to adapt the workflow to their specific requirements.

**Figure 5:**
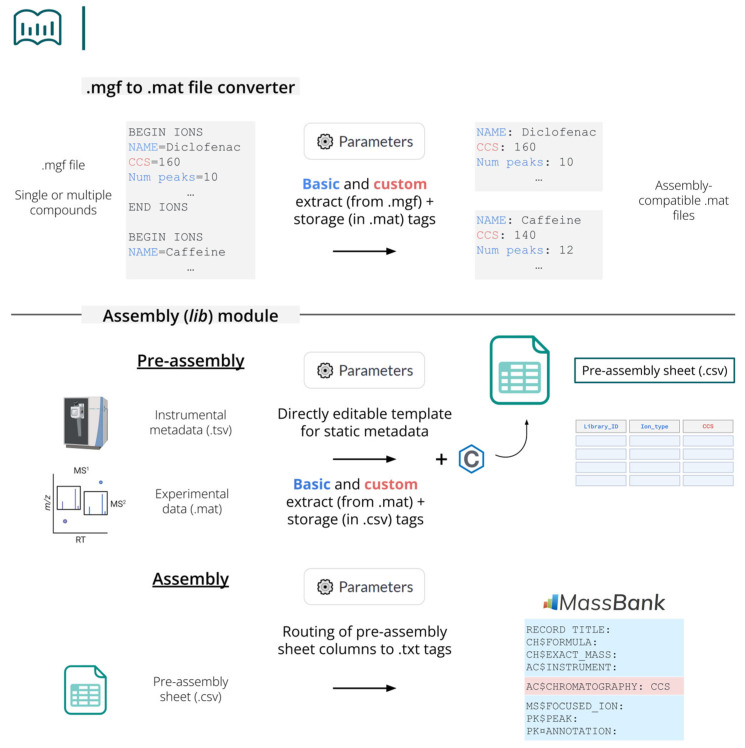
Customization options in the Librarian workflow. Users can customize the function of utilities and modules via the Parameters button. For the file converter, tags used to extract and store data can be customized by the users beyond basic extract-store pairs that are set by default (blue) to include additional tags and data fields (red). Parameters in the assembly module can be set to propagate these custom data fields to the pre-assembly sheet and onward to MassBank-format tags in the final .txt output.

A utility function is provided to convert MS^2^ .mgf files to Librarian-compatible .mat format. To perform the conversion, users upload multi-compound .mgf files which split to single .mat files per single compound, ready for use in the library assembly module. As several open-source processing software (e.g., MZmine, OpenMS) export feature-level data in .mgf format, the utility function adds flexibility in the choice of processing software. As noted above, the tags used by the converter to extract information from .mgf files, and store in the resulting .mat files, are fully customizable in the web application. Combined with the assembly customization options, users can control what data is incorporated into the final spectral records. It should be noted that users must ensure that any custom tag conforms to MassBank formatting requirements—if compliance issues arise (e.g., during record validation), the customizable parameters can be adjusted and the assembly repeated to apply the necessary corrections. An overview of the customization options is given in Figure 5.

For projects incorporating RTI information [24], Librarian provides a utility that generates .csv files formatted for direct upload to the RTI web platform [31], which performs batch conversion of recorded LC retention times to RTI values using extrapolation models based on retention data for a set of calibrant compounds. As currently implemented, the RTI platform supports conversion of up to 50 compounds per batch and requires a specific (.csv) input format. To maximize throughput, users can supply metadata (pcq sheet) and acquired MS^2^ data (including retention data) to the RTI utility, which outputs the largest possible batch-compatible .csv files. In addition, Librarian can generate inclusion lists for targeted DDA experiments. Using the mix module output sheet as input, the utility creates inclusion lists with common adducts for each mixture, with separate .csv files created for positive and negative mode. The inclusion lists are formatted for direct compatibility with acquisition software XCalibur (v 4.3; ThermoFisher Scientific, Waltham, MA USA).

## 3. Results

### 3.1. Front-End (User Interface) and Application Example

**Figure 6:**
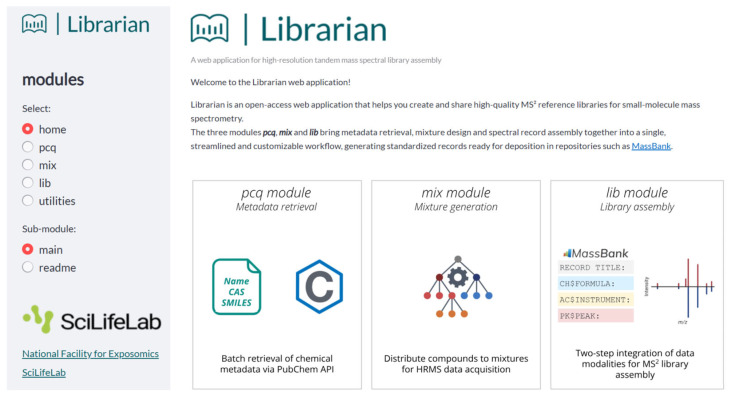
Front page of Librarian. The web application is publicly accessible via the SciLifeLab Serve platform (https://librarian.serve.scilifelab.se), and is designed to provide module functions to users directly in-browser, using a simple navigable interface to access all the modules and utilities.

**Figure 7:**
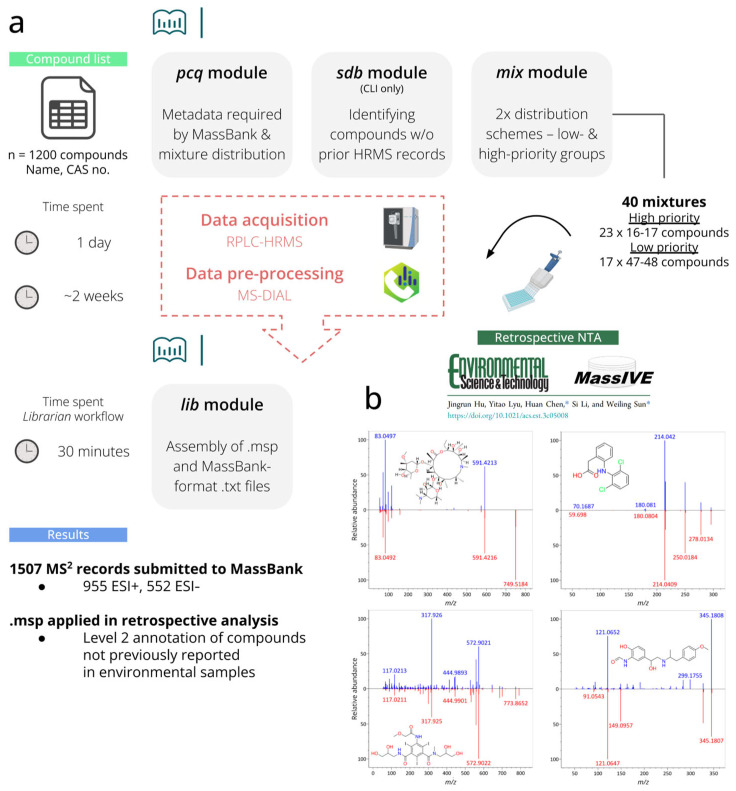
Creating and applying an LC-HRMS spectral library using Librarian. (**a**) We applied the Librarian workflow to build a spectral library for a set of 1200 pharmaceuticals, divided by acquisition priority into 23 smaller (17–18 compounds) and 17 larger (47–48 compounds) mixtures. After metadata retrieval (pcq module), survey of major repositories (sdb module, CLI only) and mixture design (mix module), in the next step of the workflow (red dashed-box) data was acquired by LC-HRMS (Orbitrap) and pre-processed using open-source software (MS-DIAL). Next, metadata and MS^2^ spectra were integrated via the Librarian assembly (lib) module, and 1507 MS^2^ records (.txt files) were submitted to MassBank. (**b**) The assembled library was used in retrospective analysis of public datasets (MassIVE) from a previous study on wastewater effluent samples from China [33]. Re-analysis recapitulated antibiotic and antifungal annotations and discovered new compound annotations from both well-known and previously unreported environmental contaminants (see Supplementary Information, Figures S2–S4 for examples, respectively). Spectral match examples shown for experimental (blue) and library (red) spectra; azithromycin (**top-left**), diclofenac (**top-right**), iopromide (**bottom-left**), formoterol (**bottom-right**).

Librarian features a simple design with a minimalistic functional style (Figure 6), allowing user-friendly, interactive interface elements including dynamic in-browser input (e.g., pcq module) and visual output (e.g., mix and lib modules). Besides providing a navigable framework for accessing Librarian modules, the interface aims for clarity and ease of use, and to be approachable for users at any level of computational experience. We demonstrate the application of Librarian to assemble an LC-HRMS spectral library of 1200 FDA-approved pharmaceuticals (Prestwick Chemical Library) [32] as part of a large in-house spectral library creation project (Figure 7) at the National Facility for Exposomics (SciLifeLab, Sweden). As a worked example for new users, a subset of this data is available for download via the web application readme submodule.

First, we compiled a list of chemical names for the 1200 pharmaceuticals that were targeted for LC-HRMS acquisition and inclusion into the new MS^2^ library. When starting the project, common names, CAS numbers and SMILES for the compounds were available from the provider, which served as the input for metadata retrieval via the pcq module. After retrieving metadata, we surveyed the public repositories (MassBank and GNPS) for any MS^2^ records already deposited to help prioritize their acquisition (Figure 7). The code for this additional step of Surveying Database (“sdb” module) is also available in the Librarian GitHub repository but can only be executed via CLI due to its dependence on large database record files (see Supplementary Information S3).

Based on then-current records (date of access: 12 January 2024), 295 out of 1200 compounds (24.6%) had no prior MS^2^ records in either database, whereas 93 (7.7%) were found deposited but only with low-resolution spectra. These 388 compounds were prioritized in the acquisition scheme and, using the mix module, distributed into 23 mixtures containing a maximum of 16–17 compounds each. The remaining 812 compounds (67.8%) which were found already deposited as HRMS records acquired with either time-of-flight (301; 25.1%) or Fourier transform-based (511; 62.9%) instruments were distributed into 17 larger mixtures (i.e., 47–48 compounds each). Based on the resulting mixture assignments, stock solutions were dispensed into 96-well plates and diluted in 70:30 water:methanol to yield final concentrations of 10 and 40 ng/mL per mixture. Standard mixtures were analyzed by RPLC coupled to HRMS (Orbitrap 480 Exploris, ThermoFisher) in both ESI+ and ESI− modes. To ensure high-quality MS^2^ acquisition, we used a targeted DDA strategy with inclusion lists created for each mixture. In addition, RTI calibrants were injected within the same sequence and later used to convert retention times for each compound to RTI values, which were incorporated in the spectral library metadata. The data acquisition method details are available in Supplementary Information S1.

The acquired HRMS data was pre-processed in MS-DIAL (v. 4.9.221218; for processing parameters, see Supplementary Information Table S1), and spectral data were exported in the .mat format, with one file exported per feature. Considering the entire workflow, pre-processing of the complete dataset was the most time-consuming step (~2 weeks), compared with the single day required for LC-HRMS acquisition, and only minutes spent using the Librarian modules. The library records were assembled using the lib module, to integrate processed HRMS data with chemical and instrumental metadata, along with RTI values and chemical ontology information (ClassyFire) into MassBank-format records (.txt) and a consolidated spectral library (.msp). Of the 1200 original compounds, 1020 (85%) were represented in the final spectral library with at least one spectral record. For the remaining 180 compounds (15%), insufficient MS/MS spectral quality or non-detection (e.g., chromatographic unsuitability and/or poor ionization efficiency) precluded creation of spectral records. In total, the project generated 1507 spectral records, of which 961 were acquired in ESI+, and 546 in ESI− mode, respectively: 463 compounds (39%) had spectral records in both ESI+ and ESI− modes, 478 compounds (40%) only in ESI+, and 79 compounds (7%) only in ESI− mode. The records were submitted to MassBank for open-access distribution and officially released on 24 October 2025 [34].

### 3.2. Environmental Monitoring Application by Retrospective NTA of Public Datasets

We applied the newly created library in retrospective NTA of publicly available LC-HRMS datasets retrieved from MassIVE [35]. However, prior to this, a practical quality control of the assembled library was performed by applying it in re-analysis of a previously published in-house dataset of river water samples from Bangladesh (Orbitrap Q Exactive HF-X, ThermoFisher) [36]. Using the new spectral library, we successfully reproduced all annotations of pharmaceuticals reported in the original study (*n* = 22; including antibiotics, anticonvulsants, antivirals, NSAIDs, etc.—annotations at confidence level 2a as per Schymanski et al. [1]; for processing parameters, see Supplementary Information Table S2). This confirmed both the quality of the acquired library spectra and the direct applicability of the Librarian output in real-world NTA workflows. Next, we applied the library to screen an independent public dataset of urban wastewater treatment plant effluents collected from two Chinese cities, Qinghai and Beijing, including one hospital effluent sample. The dataset had been acquired using a comparable LC-HRMS platform (Orbitrap Q Exactive Plus, ThermoFisher) and originally subjected to suspect and nontarget screening of antimicrobials and their transformation products [33]. Our screening resulted in a total of 113 (Level 2) spectral annotations across both ionization modes (ca. 7.5% of the assembled library), including 12 antibiotic and antifungal compounds from the original study (for examples, see Supplementary Figure S2). Moreover, the retrospective NTA workflow enabled annotation of several non-antibiotic pharmaceuticals which were not previously reported in the original study (Supplementary Figures S3 and S4), including cardiovascular drugs, NSAIDs, antidepressants, antidiabetics, and stimulants, many of which are recognized environmental contaminants. Notably, some of these annotated compounds previously lacked reference spectra in MassBank/GNPS and had little or no previous documentation of environmental occurrence (e.g., pyridostigmine, articaine, trihexyphenidyl, and formoterol; Supplementary Figure S4), highlighting how expansion of public spectral libraries can directly enable retrospective discovery of previously overlooked contaminants in NTA datasets.

### 3.3. Limitations

Several limitations of the Librarian application and workflow should be acknowledged. Librarian is not intended to provide an integrated solution for compound extraction from raw data; rather, it focuses on the downstream stages of library generation, including metadata management, record standardization, quality control and annotation, and repository-ready export. Most notably, unlike other platforms that support HRMS library creation, Librarian does not provide native raw data pre-processing and instead relies on external software (e.g., MS-DIAL, MZmine). By decoupling raw data pre-processing from library assembly, Librarian allows users to leverage established and widely adopted pre-processing tools while providing a dedicated environment for the curation and construction of high-quality spectral libraries. Finally, Librarian currently requires data uploads in specific formats and structures, which may limit compatibility and create barriers to adoption. Future development should therefore focus on expanding format compatibility and further simplifying data import workflows.

## 4. Discussion

Librarian was originally developed as an in-house workflow to meet the needs of a large-scale MS^2^ library project, whose requirements were unmet by currently available tools. We then further developed and made Librarian publicly available as a simple web application via the SciLifeLab Serve platform to provide an approachable HRMS library creation workflow option for prospective contributors. Besides providing a resource for large-scale data retrieval, mixture distribution, and record assembly, Librarian intentionally decouples from the data pre-processing workflow to be compatible with the unique software preferences of different users. Together with the extensive customization options, Librarian simplifies the compilation of structured reference records to facilitate creating, sharing, and FAIR reuse of spectral data within the broader community. Using Librarian, we compiled and deposited over 1500 MS^2^ spectra in MassBank. The complete workflow from LC-HRMS data acquisition to submission was completed in ~2 weeks, with data pre-processing accounting for most of the time spent. In comparison, library assembly using Librarian itself requires only minutes. Librarian is designed to be approachable for users at any level of computational proficiency, and owing to its simplicity and scalability, can be readily adopted for large-scale spectral library generation. As advances in data processing such as semi-automated or AI-assisted curation continue to emerge, the efficiency and speed of spectral record compilation, distribution, and reuse is also expected to dramatically increase. With the growing demand for high-quality reference spectra in metabolomics and exposomics, Librarian provides a practical and scalable solution to advance the collective efforts toward systematic exploration and mapping of the vast small-molecule spectral landscape.

## 5. Conclusions

Librarian is an open-access web application designed to streamline the creation of MS^2^ spectral libraries, allowing rapid assembly and sharing of standardized, repository-ready record. In addition to library creation, it facilitates large-scale retrieval and management of chemical identifiers and metadata, including physicochemical and toxicological information. By integrating metadata harmonization, mixture design, and record assembly into a single workflow, Librarian supports more efficient creation and dissemination of reference spectral data for metabolomics, exposomics, and environmental mass spectrometry.

## Figures and Tables

**Table 1:** Existing open-source tools supporting small molecule HRMS spectral library creation.

Feature	Librarian	RMassBank	MZmine	MS-DIAL
Primary scope	Spectral library creation (multiple formats)	Spectral library creation (MassBank format)	Pre-processing, annotation, statistical analysis, and recently library creation	Pre-processing, annotation, and statistical analysis
Target users	Contributors to MassBank and other open-access repositories	MassBank contributors	MS analysts and library contributors	MS analysts
Web-based	Yes	No	No	No
GUI	Yes	No	Yes	Yes
Installation required	No	Yes	Yes	Yes
Raw data pre-processing	Relies on external tools (e.g., MS-DIAL, MZmine)	Limited in scope, basic extraction and cleanup; advanced recalibration	Advanced	Advanced
Metadata retrieval and curation	Automated (extensive options; in-built module)	Automated (MassBank-oriented)	Automated (external; Python-based)	No
Mixture design	Automated	No	No	No
MS^2^ quality control	Automated	Automated	Automated	Manual
MS^2^ export formats	MassBank (.txt), .msp, .mgf	MassBank (.txt)	.msp, .mgf, .json	MassBank (.txt), .msp, .mgf
Batch library and metadata assembly	Yes	Yes	Yes	No

## Data Availability

The Librarian web application is accessible at SciLifeLab Serve platform (https://librarian.serve.scilifelab.se; accessed on 16 June 2026). Source code (Python) for Librarian is available through GitHub (https://github.com/jahlwe/streamlit-librarian; accessed on 16 June 2026) under MIT license. The Docker image of the hosted web application can be accessed via the container registry. All resources and documentation are provided under open licenses. Datasets used in retrospective screening are available via the MassIVE repository (Bonnefille et al.: MSV000089703-6; Hu et al.: MSV000092880).

## Supplementary Materials

The following supporting information can be downloaded at: https://www.mdpi.com/article/10.3390/metabo16060433/s1. Figure S1: Results of spectral database surveying via sdb module for the Prestwick FDA Library. Figure S2: Environmental monitoring application using public datasets. Spectral matching examples of antibiotics and antifungals reproduced by retrospective NTA. Figure S3: Environmental monitoring application using public datasets. Spectral matching examples of common pharmaceutical environmental contaminants identified by retrospective NTA. Figure S4: Environmental monitoring application using public datasets. Spectral matching examples of previously unreported pharmaceuticals identified by retrospective NTA; Table S1: MS-DIAL processing parameters for the Prestwick FDA library project. Table S2. MS-DIAL processing parameters for environmental monitoring application by retrospective NTA of public datasets. Supplementary Information S1. Prestwick FDA library project. Supplementary Information S2. PubChem query (pcq) module performance assessment. Supplementary Information S3. Description of the sdb module. Supplementary Information S4. Librarian command-line user guide.

## Abbreviations

The following abbreviations are used in this manuscript:

HRMS	High resolution mass spectrometry
NTA	Non-target analysis
LC	Liquid chromatography
ESI	Electrospray ionization
RTI	Retention time index
MS^2^	Tandem mass spectrum
DDA	Data-dependent acquisition
DBE	Double-bond equivalent
API	Application programming interface
