# Supplementary material for: Librarian: An Open-Access Web Application for High-Resolution Mass Spectral Library Assembly
Source: Metabolites. 2026 Jun 22;16(6):433. doi: 10.3390/metabo16060433 (PMC13304401; doi:10.3390/metabo16060433)
Supplement: Supplementary file 1 [file metabolites-16-00433-s001.zip › metabolites-4336461-supplementary.pdf]

Supplementary Information (SI)

Librarian: an open-access web application for high-resolution mass spectral library assembly

Jacob Ahlberg Weidenfors[1], Bénilde Bonnefille[2,3], Stefano Papazian[2,3] 🖂

[1] Unit of Integrative Metabolomics, Institute of Environmental Medicine, Karolinska Institutet, Stockholm 171 77, Sweden

[2] Science for Life Laboratory, Department of Environmental Science, Stockholm University, 114 18 Stockholm, Sweden

[3] National Facility for Exposomics, Metabolomics and Exposomics Platform, Science for Life Laboratory, Stockholm University, 171 65 Solna, Sweden

🖂 Corresponding author: Stefano Papazian stefano.papazian@aces.su.se

S1. Prestwick FDA library project.

Compound list

For the spectral library project, we used the Prestwick Chemical Libraries (PW-FDA) (1) consisting of 1,200 compounds representing a broad range of small molecules with medical applications, such as pharmaceuticals and diagnostic reagents, the majority of which received current or previous approval by the FDA and/or the EMA for at least one use or indication. The compounds were obtained from the Compound Center of Chemical Biology Consortium Sweden (CBCS), SciLifeLab Campus Solna (Stockholm, Sweden). Using the *mix* module of Librarian, the PW-FDA compounds were distributed into 40 different mixtures. For each compound in each mixture, expected accurate masses, ion species, and charged adducts for each ionization mode were generated automatically by the *mix* module. These outputs were subsequently compiled by the Librarian *ddaLists* utility into a targeted inclusion list compatible with Xcalibur.

Instrumental acquisition

Each standard mixture was analyzed by LC-HRMS using a Vanquish Horizon system coupled to an Orbitrap Exploris 480 (ThermoFisher). Data were acquired in both ESI+ and ESI–, with each mixture injected once per ESI mode. The HRMS instrument was operated in parallel MS^1 full-scan (80-950 m/z; 120,000 nominal resolution at m/z 200) and MS^2 data-dependent acquisition (DDA) (30,000 nominal resolution at m/z 200). From each MS^1, the top 7-10 (top-N) most intense precursors were

selected for fragmentation, with a fallback rule triggering fragmentation of the most intense ion when none of the inclusion-list masses were detected. For each ionization mode, 20 µL of each mixture was injected onto a reversed phase column (Acquity UPLC BEH C18, 1.7 µm, 2.1 × 100 mm, 130 Å, Waters) maintained at 50°C. Separation was performed using a binary gradient at 0.4 mL/min with the mobile phases (A) water (Optima LC-MS grade, Fisher Chemical) containing 1 mM ammonium fluoride (Honeywell Fluka, ≥98.0%) and (B) 100% methanol (Optima LC-MS grade, Fisher Chemical).

Data pre-processing

Following acquisition, raw LC-HRMS files (.raw) were converted to .abf format (Reifycs ABF Converter) and pre-processed with the open-source software MS-DIAL (v4.9.221218). After peak picking, features were filtered by matching accurate masses to PW-FDA ion masses, retaining only features with acquired MS^2 spectra, and applying blank filtering. The processed data was exported in .mat format, with one file exported per compound feature. Complete pre-processing parameters for MS-DIAL are provided in S2.

Retention time indexing

To facilitate inter-laboratory comparison of PW-FDA compound retention times, retention time indexing (RTI) was implemented following the approach by Aalizadeh et al (2). During LC-HRMS acquisition, mixtures of RTI calibrants for ESI+ and ESI- modes were injected within the same injection sequence of the PW-FDA compounds. Raw data from RTI injections were processed as described above, and the measured retention times of both RTI calibrants and PW-FDA compounds were submitted to the RTI web application. Calibration curves were generated using the "*Auto-calibrate*" option, and RTI values were obtained by providing input sheets containing raw PW-FDA retention times and compound SMILES.

S2. PubChem query (pcq) module performance assessment

To benchmark the pcq module performance, we manually collected and curated correct reference CIDs for the 1,200 compounds of our original library expansion project and compared these to CIDs retrieved directly using names, CAS registry numbers and SMILES obtained from the supplier, respectively. Retrieved CIDs not matching the curated CID for each compound and query type were investigated manually and assessed for the type and potential source of error. Mismatches between reference and retrieved CIDs where the retrieved CID represented the same compound in either of the keto-enol forms, or where stereochemistry was unspecified were not considered query errors.

A total of 15 errors (1.2%, 15 of 1,200) were encountered for queries using compound names from the supplier. Six failed queries related to bracketed elements within the name string (e.g, "Hydroxytacrine maleate (R,S)") which fail to be resolved in compound API requests (but were resolved in a direct web-based PubChem search). Three failed queries were for large, complex or multicomponent chemical entities (ivermectin, verteporfin, tyloxapol; "No discrete structure" annotation in PubChem web-searches) which did not resolve a CID in compound API requests. Two failed queries concerned salts (oxantel and pyrvinium pamoate) and were related to a limitation in the Librarian code. For salt queries, if a parent compound annotation is missing in the PubChem entry being read, Librarian defaults to desalting the compound by creating an RDKit mol object from the available SMILES and extracting the largest fragment of the multicomponent mol object (rdMolStandardize.LargestFragmentChooser() function). In the case of these compounds, the pamoate moiety is larger than the active moieties, resulting in incorrect metadata retrieval. Four failed queries were related to misspelled compound names.

A total of 10 errors (0.8%, 10 of 1,195 — 5 compounds lacking a CAS registry number from the supplier) were encountered for queries using CAS registry numbers from the supplier. The three errors relating to large multicomponent structures (ivermectin, verteporfin, tyloxapol) and two errors desalting fallback errors (oxantel and pyrvinium pamoate) seen for name queries were reproduced in CAS registry number queries, and an additional two errors relating to large ("No discrete structure") entities were encountered (rifabutin and methylbenzethonium chloride). Two CAS registry number queries failed to resolve a CID through compound API request (2984-97-0 for 6,7-dimethoxy-1-methyl-1,2,3,4-tetrahydroisoquinoline, and 32872-77-1 for (R)-butoconazole (but were resolved in PubChem web searches). One error was related to desalting of an organomercury compound (merbromin) where the supplied CAS registry number (129-16-8) resolved to a CID (102248350) where the mercury atom was treated as a counterion rather than a structural component, resulting in its abstraction and re-query of the remaining structure by the Librarian code.

A total of 98 errors (8.2%, 98 of 1,200 were encountered for queries using SMILES strings from the supplier. While only recapitulating two of the errors for large multicomponent structures (rifabutin and tyloxapol) as well as the desalting fallback errors (oxantel and pyrvinium pamoate), the majority of errors (93 of 98) were related to improper SMILES notation which either failed to resolve, or resolved CIDs for non-identical structures. A common error (n=58) related to non-equivalent, improper positioning (e.g., causing breaking-up of resonance structures) of double bonds in the

supplied SMILES. Other common errors related to improper notations of protonation (n=9) and absent or excessive double bonds (n=8). While offering a greater degree of specificity, our results underscore a greater risk for errors, as well as a relatively greater impact of input errors for the more complex and precise SMILES queries relative to e.g. name queries.

S3. Description of the sdb module

During the initial development of Librarian a module was written to survey records of the spectral repositories MassBank and GNPS. Because the module functions require access to large database record files, it could not be adapted for the Librarian web application but may be accessed by cloning the streamlit-librarian GitHub repository for use via the CLI.

The sdb module provides functions to interrogate MassBank and GNPS records for the presence of records and gather information about the types of records present.

The module accepts a pcq output sheet as input, indexes repository data files for fast lookup of compound records and returns the input sheet with added survey results. For each compound in the input list, the module returns the number of existing entries present, their accession numbers, the ionization modes and the instrument types with which the spectral data of entries were recorded.

To survey MassBank, sdb accepts records in .json format. To survey GNPS, .csv and .mgf formats are accepted. The records must be downloaded manually from the respective repository.

The sdb module was used during our original spectral library project to help prioritize acquisition of the 1,200-large compound library (see fig. S1 below).

S4. Librarian command-line user guide

A brief user guide for the command-line distribution of Librarian is given below. We recommend working with Librarian through a dedicated virtual environment (e.g., conda) to simplify installation and management of necessary dependencies.

First, the Librarian command-line GitHub repository should be cloned to your local system and necessary dependencies installed. Once cloned, navigate to the main Librarian folder through your command-line interface (CLI) to access module functions via the “main” Librarian script file.

Module functions are accessed by standard Python CLI syntax, by supplying commands which are read by the main script file *librarian.py*. I.e., all commands or calls to use module functions are initiated with the terms *python librarian.py* followed by the abbreviation for a specific module and

related arguments. Brief descriptions, commands and arguments for use of each module and utility are given below.

The pcq module initiates the Librarian workflow. Use via the command-line requires only a .csv file preferably placed in the input folder. Arguments and a use example are given below.

Module	***Argument***	***Description***
pcq		
	**[sheet_path]**	Path to input compound list. E.g., "input/compoundList.csv".
	**[output_path]**	Specifies a save path for the pcq output.
	-c [canonicalize_smiles]	Canonicalizes the input in SMILES queries via RDKit.
	-d [drop_stereochemistry]	Drops stereochemistry from canonicalized SMILES. Requires -c.
	Use example	python librarian.py pcq input/compoundList.csv output/pcq_out.csv

The sdb module may be used to interrogate public repository records for compound entries. Use via the command-line requires that at least one repository record file is downloaded and placed within the files/survey folder. Several repositories may be interrogated at a time (i.e., with one command prompt) or separately. Arguments and a use example are given below.

Module	***Argument***	***Description***
sdb		
	**[sheet_path]**	Path to an output sheet from the pcq module. E.g., "output/pcq_out.csv".
	**[output_path]**	Specifies a save path for the sdb output.
	-mb [file_name]	Specifies file names for the repository records to be surveyed. All

	-cp [file_name]<br>-cn [file_name]<br>-mp [file_name]<br>-mn [file_name]	downloaded records should be placed in the files/survey folder. Only the name of the file needs to be supplied. The -mb should be followed by the file name of the MassBank records, -cp and -cn by file names of GNPS records in .csv formats in positive and negative mode respectively, -mp and -mp by file names of GNPS records in .mgf formats in positive and negative modes respectively. At least one flag and file name should be provided.
	Use example	python librarian.py sdb pcq_out.csv sdb_out.csv -mb MassBank.json -cp GNPS_pos.csv -cn GNPS_neg.csv

The mix module may be used to distribute compounds to mixtures following metadata retrieval. Use via the command-line requires an output sheet from the pcq or sdb modules (the pcq-related metadata is the only required information – sdb output will have that, too). Use also requires an argument for the desired number of mixtures. Options are available to auto-assign compounds if the algorithm fails to find a solution conforming to the minimum mass difference within mixtures for the compounds and desired number of mixtures. Arguments and a use example are given below.

Module	***Argument***	***Description***
mix		
	**[sheet_path]**	Path to an output sheet from pcq or sdb. E.g., "output/pcq_out.csv".
	**[n_mixes]**	Desired number of mixtures, integer.
	**[output_path]**	Specifies a save path for the mix output.
	-d [min_diff]	Desired minimum mass difference within mixtures in Da. Defaults to 0.01.
	-a [True/False]	Auto-assign unassigned (by mass) compounds by xlogp. Defaults to True.
	Use example	python librarian.py mix output/pcq_out.csv 10 output/mix_out.csv -d 0.05 -a False

The pre-assembly module ("precomp" in the command-line version) is used to compile required data and optional data sources in preparation for the final library assembly. Use via the command-line requires an output sheet with pcq-derived chemical metadata, a .tsv file with experimental

parameters placed in the files/compiler folder as well as the presence of .mat files inside “pos” or “neg” folders (preceded by any optional folder structure) in the input/mat folder. The ionization mode should be provided as an argument.

Optional data types (RTI, ClassyFire) can be included by placing files in their respective folder, inside the input folder. Sheets from the RTI web application should be placed in the input/RTI folder, inside either “pos” or “neg” folders. A sheet with ClassyFire data from the ClassyFire Batch website (4) should be placed inside the input/classyfire folder and be named “cf_manual”. Arguments and a use example are given below. To be noted, ClassyFire chemical ontologies are automatically provided by MassBank for new records since release 2025.05.1.

Module	Argument	Description
precomp		
	**[mode]**	Ionization mode of data being (pre-)pre-compiled. "pos" or "neg".
	**[data_dir]**	Path to folder containing folders with .mat files. (which should be organized by mode in “pos” and “neg” folders respectively)
	**[ref_path]**	Path to a sheet with metadata from the pcq module.
	**[tsv_path]**	Path to static (author, instrumental, e.g.) metadata .tsv file.
	**[output_path]**	Specifies a save path for the pre-assembly sheet.
	-fa [True/False]	Perform fragment annotation. Defaults to True.
	-p [float]	Mass deviation tolerance in ppm for fragment annotation. Default = 10.
	-rti [True/False]	Specifies a folder to look for RTI web app .csv output sheets, if applicable. Requires that the sheets are separated into subfolders “pos” and “neg” for the respective modes.
	-cf	Specifies a path to a sheet with ClassyFire chemical ontology data from the ClassyFire Batch website.
	Use example	python librarian.py precomp pos input/mat_files pcq_out.csv static_metadata.tsv output/preAssembly_pos.csv -fa -p 8 -rti input/RTI_data

The assembly module (“compile” in the command-line version) is used to generate MassBank-format .txt files and .msp library files from the pre-assembly module output sheet. The pre-assembly sheet may be manually edited prior to final assembly to e.g. exclude particular compounds or append additional data. Use via the command-line requires the presence of a pre-assembly output sheet (named either preComp_pos or _neg) to be present in the output/compiler folder. A start for record accession numbering and the ionization mode should be provided as arguments.
Arguments and a use example are given below.

Module	Argument	Description
compile		
	**[sheet_path]**	Path to a pre-assembly sheet.
	**[output_dir]**	Folder to which the assembled library output is sent.
	**[acc_long]**	Full accession prefix. (format MSBNK-USERID-SHORTACC)
	**[acc_short]**	Short accession prefix.
	**[acc_start]**	Start of accession numbering.
	**[mode]**	Ionization mode of data being compiled. "pos" or "neg".
	Use example	python librarian.py compile 123 pos

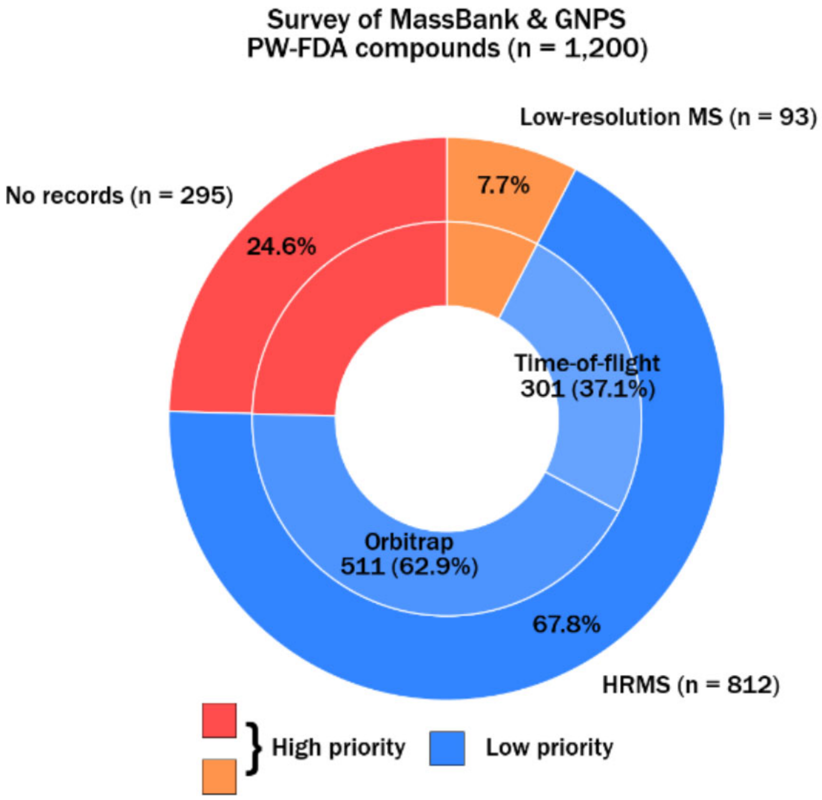

Figure S1. Results of spectral database surveying via *sdb* module for the Prestwick FDA Library. The sdb module was used to interrogate repository records (accessed 2024-12-01) for prior entries to guide mixture distribution and prioritize compounds for acquisition during our project.

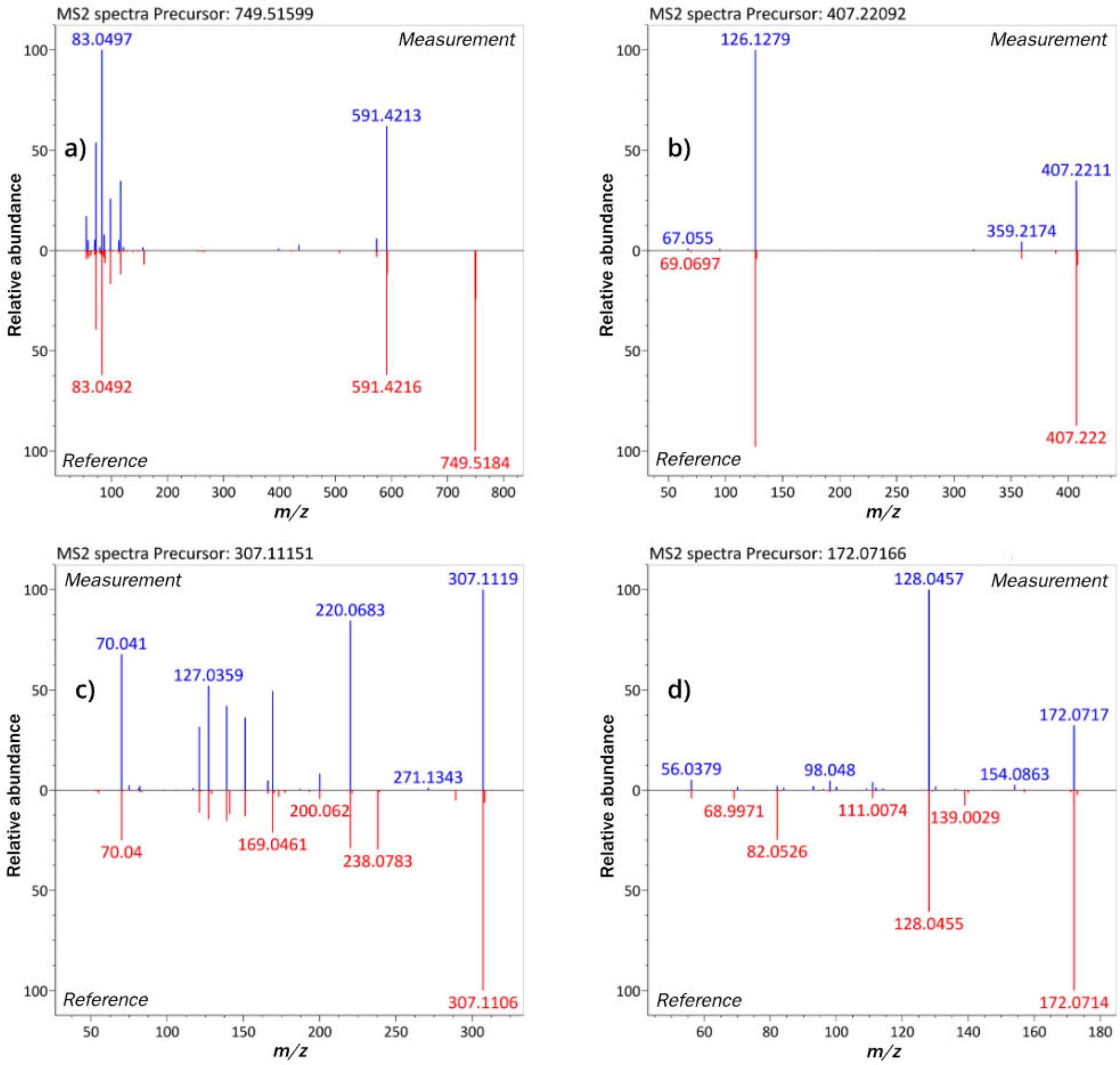

Figure S2. Environmental monitoring application using public datasets. Spectral matching examples of antibiotics and antifungals reproduced by retrospective NTA**.** Retrospective analysis of wastewater treatment plant effluents from two Chinese cities (Qinghai and Beijing) (3) using the PW-FDA MS^2 libraries assembled via Librarian. The analysis reproduced confident annotations (Level 2) for all the antibiotics and antifungals reported in the original study and present in the current library, including a) azithromycin, b) lincomycin, c) fluconazole and d) metronidazole.

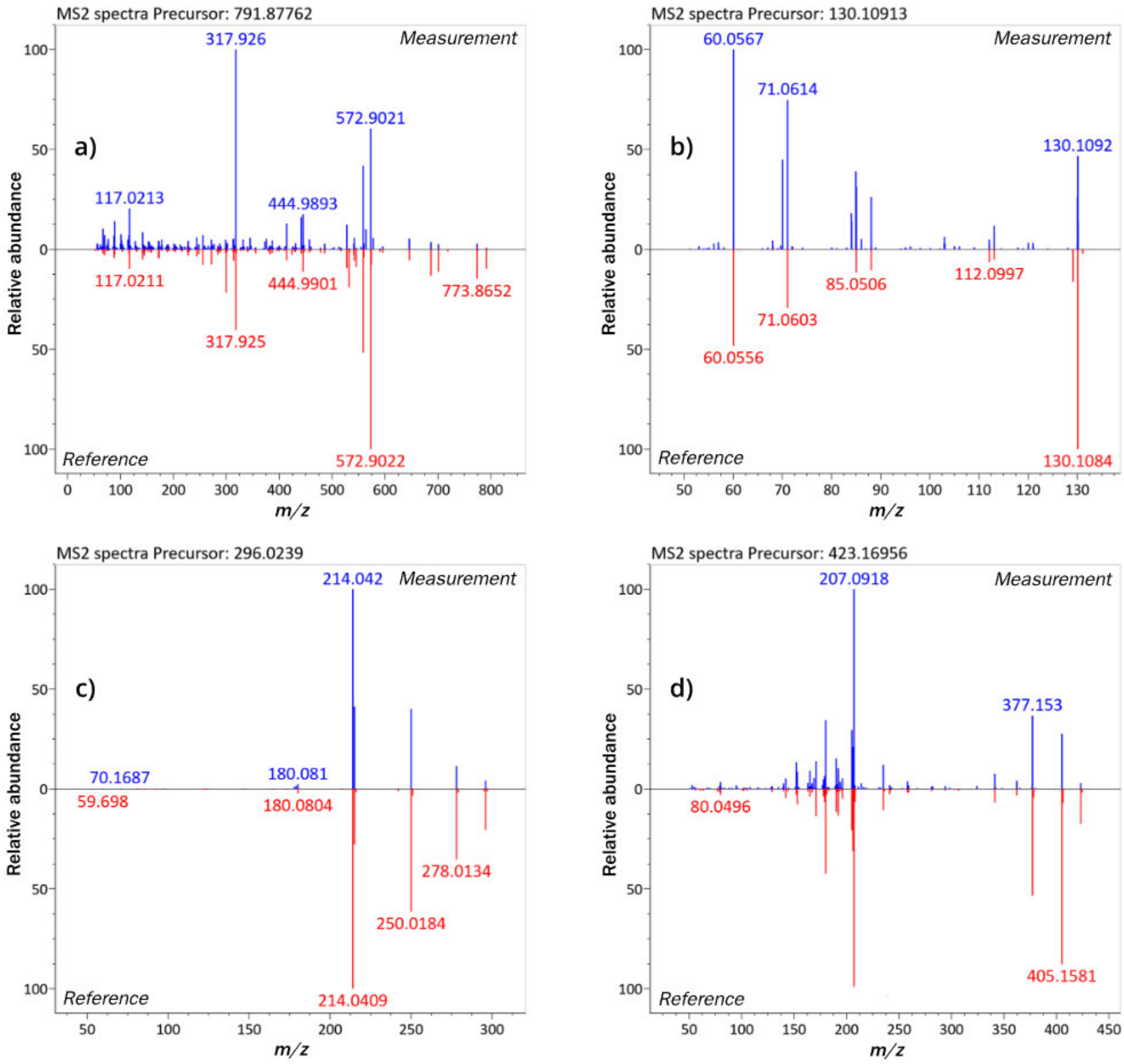

Figure S3. Environmental monitoring application using public datasets. Spectral matching examples of common pharmaceutical environmental contaminants identified by retrospective NTA. Retrospective analysis of wastewater treatment plant effluents from two Chinese cities (Qinghai and Beijing) (3) using the PW-FDA MS^2 libraries assembled via Librarian. Several confident annotations (Level 2) were attained of non-antibiotic pharmaceuticals including a) the radiological contrast agent iopromide, b) the antidiabetic drug metformin, c) the non-steroidal anti-inflammatory drug diclofenac, and d) the antihypertensive drug losartan. Iopromide was identified exclusively in effluent samples collected from the hospital wastewater treatment plant in Beijing.

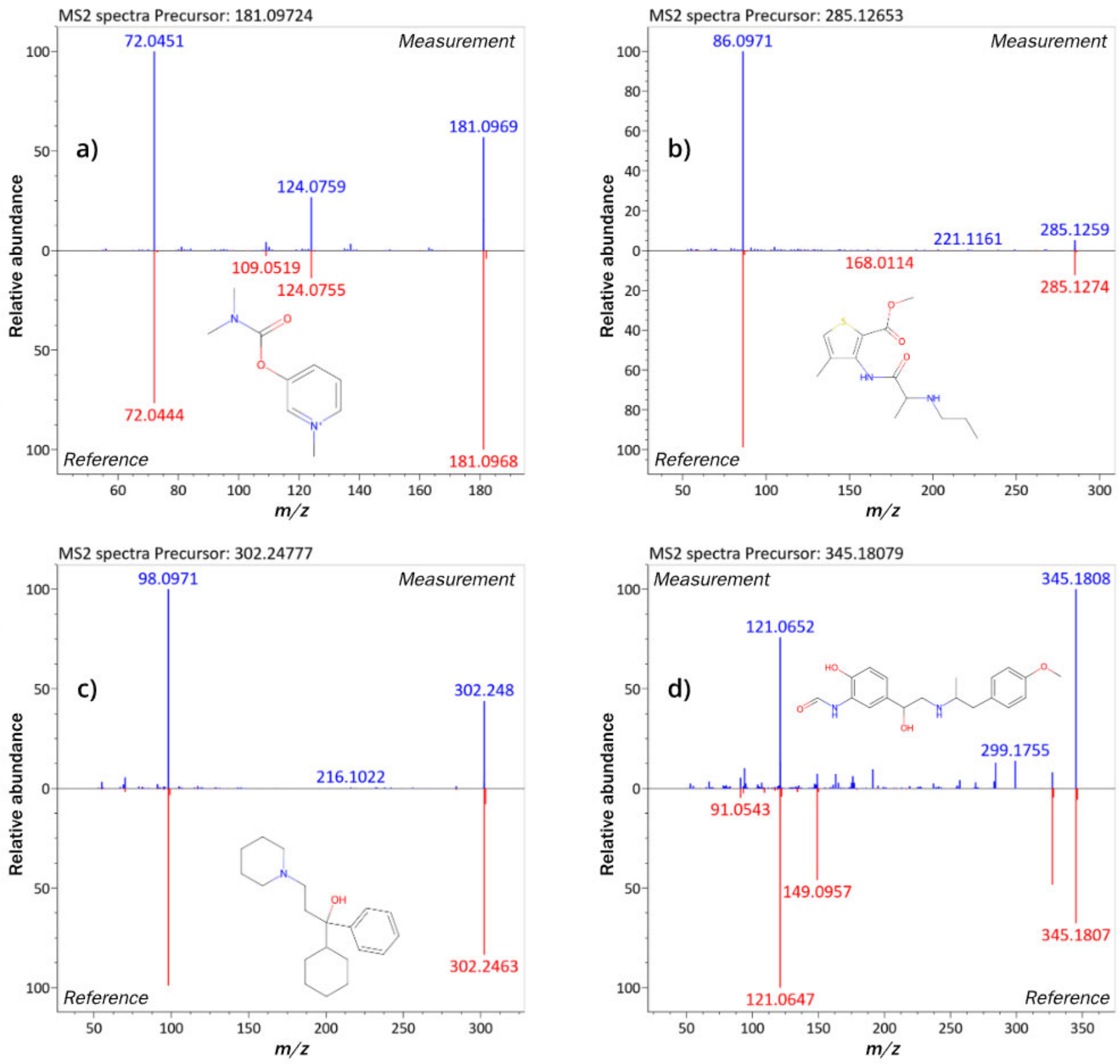

Figure S4. Environmental monitoring application using public datasets. Spectral matching examples of previously unreported pharmaceuticals identified by retrospective NTA. Retrospective analysis of wastewater treatment plant effluents from two Chinese cities (Qinghai and Beijing) (3) using the PW-FDA MS^2 libraries assembled via Librarian. Additional annotations for compounds at confidence Level 2 for novel pharmaceuticals which were not previously deposited in public spectral databases (MassBank and GNPS) and with limited prior documentation of environmental occurrence. Spectral matches and chemical structures shown for a) the cholinergic agent pyridostigmine, b) the local anaesthetic articaine, c) the antimuscarinic trihexyphenidyl and d) the β2 receptor agonist formoterol.

Supplementary Table S1. MS-DIAL processing parameters for the Prestwick FDA library project

Data collection		Peak detection	
MS1 tolerance	0.002	Minimum peak height	90 000
MS2 tolerance	0.005	Mass slice width	0.05
Maximum charge n	3	*Advanced settings*	
Consider Cl and Br elements	TRUE	Smoothing method	Linear weighted moving average
		Smoothing level	3
		Minimum peak width	5
**Identification**		**Adducts**	
As RTs of compounds were unknown upon injection, all identifications were made without consideration of retention time through supplied lists of ion types/adducts with accurate mass information (one list per mix and mode)		ESI+: M+H+, M+NH4+, M+Na+, M+H-H2O+, M+H-2H2O+, 2M+H+, M+2H2+<br>ESI–: M-H-, M-H2O-H-, M+Cl-, 2M-H-, M-2H2-	
**Alignment**			
Reference file	Either of duplicated .abf files per mixture	Remove features based on blank information	TRUE
RT tolerance (min)	0.2	Sample max / blank average	5 fold-change
MS1 tolerance	0.005	Keep 'reference matched' metabolite features	FALSE
Advanced settings		Keep 'suggested (w/o MS2)' metabolite features	FALSE
RT factor	0.5	Keep removable features and assign the tag	TRUE
MS1 factor	0.5	Gap filling by compulsion	TRUE

Supplementary Table S2. MS-DIAL processing parameters for environmental monitoring application by retrospective NTA of public datasets.

**Data collection**		**Peak detection**	
MS1 tolerance	0.001	Minimum peak height	50 000
MS2 tolerance	0.005	Mass slice width	0.05
Maximum charge n	2	*Advanced settings*	
Consider Cl and Br elements	TRUE	Smoothing method	Linear weighted moving average
		Smoothing level	3
		Minimum peak width	5
**Identification**		**Adducts**	
MSP file	PWFDA .msp	ESI+: M+H+, M+NH4+, M+Na+, M+H-H2O+, M+H-2H2O+, 2M+H+, M+2H2+	
Retention time tolerance		ESI–: M-H-, M-H2O-H-, M+Cl-, 2M-H-, M-2H2-	
Accurate mass tolerance (MS1)	0.01		
Accurate mass tolerance (MS2)	0.05		
Identification score cutoff	70%		
Use RT for scoring, filtering	FALSE		
**Alignment**			
Reference file	Largest file by byte size (B2-3)	Remove features based on blank information	TRUE
RT tolerance (min)	0.3	Sample max / blank average	5 fold-change
MS1 tolerance	0.005	Keep 'reference matched' metabolite features	FALSE
Advanced settings		Keep 'suggested (w/o MS2)' metabolite features	FALSE
RT factor	0.5	Keep removable features and assign the tag	TRUE
MS1 factor	0.5	Gap filling by compulsion	TRUE
